# Sorting Nexin 10 as a Key Regulator of Membrane Trafficking in Bone-Resorbing Osteoclasts: Lessons Learned From Osteopetrosis

**DOI:** 10.3389/fcell.2021.671210

**Published:** 2021-05-20

**Authors:** Ari Elson, Merle Stein, Grace Rabie, Maayan Barnea-Zohar, Sabina Winograd-Katz, Nina Reuven, Moran Shalev, Juraj Sekeres, Moien Kanaan, Jan Tuckermann, Benjamin Geiger

**Affiliations:** ^1^Department of Molecular Genetics, The Weizmann Institute of Science, Rehovot, Israel; ^2^Institute of Comparative Molecular Endocrinology, University of Ulm, Ulm, Germany; ^3^Hereditary Research Laboratory and Department of Life Sciences, Bethlehem University, Bethlehem, Palestine; ^4^Department of Immunology, The Weizmann Institute of Science, Rehovot, Israel

**Keywords:** osteoclast, osteopetrosis, ARO, sorting nexin, SNX10, bone resorption

## Abstract

Bone homeostasis is a complex, multi-step process, which is based primarily on a tightly orchestrated interplay between bone formation and bone resorption that is executed by osteoblasts and osteoclasts (OCLs), respectively. The essential physiological balance between these cells is maintained and controlled at multiple levels, ranging from regulated gene expression to endocrine signals, yet the underlying cellular and molecular mechanisms are still poorly understood. One approach for deciphering the mechanisms that regulate bone homeostasis is the characterization of relevant pathological states in which this balance is disturbed. In this article we describe one such “error of nature,” namely the development of acute recessive osteopetrosis (ARO) in humans that is caused by mutations in sorting nexin 10 (SNX10) that affect OCL functioning. We hypothesize here that, by virtue of its specific roles in vesicular trafficking, SNX10 serves as a key selective regulator of the composition of diverse membrane compartments in OCLs, thereby affecting critical processes in the sequence of events that link the plasma membrane with formation of the ruffled border and with extracellular acidification. As a result, SNX10 determines multiple features of these cells either directly or, as in regulation of cell-cell fusion, indirectly. This hypothesis is further supported by the similarities between the cellular defects observed in OCLs form various models of ARO, induced by mutations in SNX10 and in other genes, which suggest that mutations in the known ARO-associated genes act by disrupting the same plasma membrane-to-ruffled border axis, albeit to different degrees. In this article, we describe the population genetics and spread of the original arginine-to-glutamine mutation at position 51 (R51Q) in SNX10 in the Palestinian community. We further review recent studies, conducted in animal and cellular model systems, that highlight the essential roles of SNX10 in critical membrane functions in OCLs, and discuss possible future research directions that are needed for challenging or substantiating our hypothesis.

## Introduction

### The Molecular Pathways That Regulate the Development and Function of Osteoclasts

Bone formation and homeostasis are tightly regulated processes, which depend on the concerted action of three major cell types: mesenchyme-derived OBs, which build bone, hematopoietic OCLs that degrade it, and osteocytes, which are also of mesenchymal origin and act as ‘bone mechanosensors’ that orchestrate the interplay between the former two cell types (Feng and Teitelbaum, 2013; Robling and Bonewald, 2020). The activities of OCLs and OBs are highly coordinated in time and space, leading to the replacement of old and damaged bone with new and intact tissue. This process of bone remodeling, in which OB and OCL activities are coordinated, removes micro-cracks and other structural defects that develop with time and maintains the physiological mass and physical properties of the bone matrix. Bones can be shaped or re-shaped also through the independent actions of these two cell types (Seeman, 2009; Feng and McDonald, 2011; Langdahl et al., 2016). Overall, bone production and degradation are well-regulated and balanced, and disruption of this balance can alter bone mass and cause serious disease.

**FIGURE 1:**
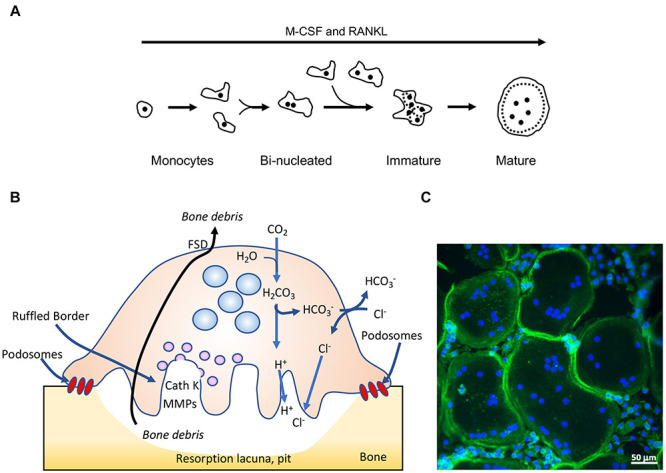
Osteoclasts: form and function. **(A)** Outline of osteoclastogenesis in culture. M-CSF and RANKL induce mononucleated monocytes to differentiate and fuse to form bi-nucleated cells, which continue to fuse and form immature, multi-nucleated cells characterized by their random organization of podosomes (small black dots). The immature cells then mature and become rounder, and display a podosomal sealing zone-like belt at their periphery. **(B)** Schematic drawing of a mature, bone-resorbing osteoclast. OCLs adhere to bone using podosomes, actin-rich adhesion structures that, when grown on mineralized matrix, form a closed array (sealing zone) that isolates the area of bone that is to be degraded. The cells then secrete from their ventral ruffled border membrane protons, which dissolve the mineral components of the bone matrix, and proteases [such as cathepsin K (CathK) and matrix metalloproteases (MMPs)] that degrade its protein components. This combined activity results in formation of a resorption lacuna, or pit, in the bone surface. OCLs then endocytose the bone degradation products through their ruffled border membrane, transport them through the cell, and secrete them from the apical Functional Secretory Domain (FSD). **(C)** Mature mouse OCLs grown on glass coverslips, stained for actin (green, highlights the closed podosomal sealing zone-like array) and DNA (blue, nuclei). Note much smaller cells that have not fused and are located between the larger OCLs. Panels **(B,C)** were modified with permission from Shalev and Elson (2018).

Osteoclasts are giant, multinucleated phagocytes that originate in monocyte precursor cells. The cytokines M-CSF and RANKL trigger a complex and multi-stage process, in which mono-nuclear monocytes are induced to differentiate and fuse to form multi-nucleated cells that then differentiate further into mature bone-resorbing OCLs (Teitelbaum, 2007) (Figure 1A). It has been shown recently that mature OCLs can undergo fission *in vivo*, generating smaller multi-nucleated “osteomorphs” that can re-enter the fusion process and participate once again in forming mature OCLs (McDonald et al., 2021). Bone resorption is initiated when OCLs adhere to mineralized tissue via podosomes, specialized punctate integrin-based actin-rich adhesions that assemble into a peripheral belt-shaped array (also called a SZ) that confines the bone area destined for degradation (Figures 1B,C). Secretory lysosomes then fuse with the area of the ventral cell membrane that is delimited by the SZ to form a ‘RB,’ which is enriched with proton pumps and chloride transporters that drive the acidification of the OCL-bone interface. Fusion of lysosomes with the RB also discharges cathepsin K and other proteolytic enzymes onto the underlying bone surface. The combination of low pH and proteases leads to localized dissolution of the mineral and organic components of the bone matrix, and generates a resorption cavity (Novack and Teitelbaum, 2008; Teitelbaum, 2011). OCLs then endocytose the bone matrix debris from the resorption cavity through a specialized region of the RB. The debris are transported through the cell body to a specialized Functional Secretory Domain (FSD) located on the dorsal plasma membrane, through which the debris are secreted (Nesbitt and Horton, 1997; Salo et al., 1997; Mulari et al., 2003) (Figure 1B). Mature, functional OCLs are therefore polarized cells, in which directional transport takes place to and from their distinct dorsal and ventral membranes.

The process whereby uncommitted monocytes develop into mature, active OCLs includes several distinct stages that are driven by M-CSF, which promotes the survival and proliferation of the monocyte OCL precursor cells, and RANKL, which provides the major pro-osteoclastogenic signals. The molecular events and transcriptional programs that are induced by both cytokines are essential and sufficient for inducing monocyte precursor cells to differentiate into mature, bone-resorbing OCLs (Feng and Teitelbaum, 2013; Tsukasaki et al., 2020). Specifically, M-CSF activates its receptor tyrosine kinase, c-Fms, which, in turn, activates ERK1/2, PI3K, and Src signaling. Binding of RANKL to its receptor, RANK, induces the receptor to bind TNF Receptor-Associated Factors (TRAFs), which then activate the ERK, NFkB, and PI3K pathways and ultimately induce expression of NFATc1, the major fate-determining osteoclastogenic transcription factor (Wada et al., 2006; Feng and Teitelbaum, 2013; Takayanagi, 2021). Sustained activation of NFATc1 also requires its dephosphorylation by calcineurin, whose Ca^++^-dependent activity requires signaling by the OSCAR and TREM2 receptors and their downstream ITAM-motif containing effectors DAP12 and FϵRI (Zou and Teitelbaum, 2015; Humphrey and Nakamura, 2016). Finally, formation of resorption-competent OCLs also requires major cytoskeletal and membrane trafficking processes that act together to construct the resorptive apparatus of the cells. Attachment of OCLs to mineralized matrix is mediated by heterodimeric integrins, chiefly consisting of the αvβ3 chains. Binding of this integrin to RGD motifs in extracellular matrix proteins activates the Src tyrosine kinase, which leads to activation of Syk, Vav3, and the Rho-family GTPases Rac1 and Rac2. These integrin-driven signaling events ultimately lead to reorganization of the actin-based cytoskeleton and assembly of podosomes into SZs, enable lysosome fusion and formation of the RB, and induce OCL polarization (Feng and Teitelbaum, 2013; Georgess et al., 2014; Linder and Wiesner, 2016).

Fine spatial and temporal tuning of membrane composition, compartmentalization, and dynamics play essential roles throughout the formation and development of OCLs, starting with the early stimulation of monocytes by M-CSF and RANKL and culminating in full OCL maturation and activation. This is evident in the extensive cell-cell fusion that occurs throughout osteoclastogenesis, until OCLs gain full resorption competence and stop fusing with other mature OCLs (Barnea-Zohar et al., 2021). It is also evident in the ability of OCLs to sense the properties of the underlying bone surface and to translate this information into cues that drive SZ formation at specific locations of the ventral cell membrane. The RB is then formed at these locations by another membranal event, namely the fusion of secretory lysosomes with the cell membrane. Membrane-related events also drive secretion of protons and proteases that degrade the underlying bone, endocytosis of bone matrix debris and their ultimate secretion via the dorsal FSD, leading to cell polarization (Feng and Teitelbaum, 2013; Georgess et al., 2014; Linder and Wiesner, 2016). That said, the nature of the molecular regulators of these processes is still unclear. We propose here that SNX10, which belongs to a large protein family that regulates vesicular trafficking in cells, plays a central role in spatially and temporally regulating the composition of diverse OCL membrane compartments. We further hypothesize that by doing so, SNX10 controls and affects membrane trafficking along the axis that links the plasma membrane with the RB, including processes, such as cell fusion and RB formation, in which it apparently plays an indirect role. We further propose that mutations in other known ARO-associated genes also disrupt the plasma membrane-to-RB axis, leading to related phenotypes. Here we examine this hypothesis in the context of a mutant of SNX10 that is associated with the genetic disease ARO.

### Learning About Osteoclasts From a Genetic Disease: The Case of Autosomal Recessive Osteopetrosis (ARO)

Decreased OCL-mediated bone resorption can occur as a result of genetic mutations that suppress the production of OCLs or inhibit their activity. Of particular importance in this context are genetic diseases whose mode of inheritance is autosomal recessive (AR), which allow linking individual genes and proteins with specific aspects of osteoclastogenesis or OCL function in a non-hypothesis driven, “real world” setting. Reduced bone resorption can disrupt the balance between bone formation and degradation and may lead to osteopetrosis, a heterogenous group of rare genetic diseases that are characterized by mild to life-threatening increases in bone mass. Osteopetrosis was initially described as “Marble Bone Disease” at the beginning of the 20th century by Albers-Schoenberg. This form of osteopetrosis is inherited in an autosomal dominant manner, its symptoms are typically first detected in adults, and its progression is relatively benign (Balemans et al., 2005; Bollerslev et al., 2013). Another form of this disease is ARO, also known as malignant infantile osteopetrosis due to the tendency of its symptoms to become progressively worse. ARO is a rare disease with a global incidence of 1 in 250,000 live births, and is lethal unless treated by HSCT as noted further below (Sobacchi et al., 2013; Palagano et al., 2018). In ARO the trabecular pattern of the bone is lost and the differentiation between cortex and medulla is largely missing. Consequently, patients suffer from bone marrow failure as a result of bony encroachment into the hematopoietic niche, which leads to consequent pancytopenia and hepatosplenomegaly (Pangrazio et al., 2013). Skull changes may lead to hydrocephalus, choanal stenosis, and nerve compression, which can result in blindness, hearing loss, and cranial nerve palsies (Balemans et al., 2005). Additional symptoms include micrognathia, small thorax, pathological fractures, hypocalcemia, and severe dental defects as well as osteomyelitis of the jaw. Patients with more severe variants of this disease typically present also with severe failure to thrive during early infancy and do not survive past their first decade of life (Penna et al., 2019).

Most cases of ARO are caused by the presence of inactive OCLs [“OCL-rich ARO,” (Sobacchi et al., 2013)] due to mutations in *TCIRG1, CLCN7*, or *OSTM1*, which encode components of the proton pump or Cl^–^/H^+^ antiporter of OCLs that are essential for acid secretion. Mutations in *PLEKHM1*, whose lysosome-associated protein product participates in vesicular trafficking, are another cause of this variant of ARO (Li et al., 1999; Frattini et al., 2000; Kornak et al., 2000, 2001; Chalhoub et al., 2003; Quarello et al., 2004; Van Wesenbeeck et al., 2007; Del Fattore et al., 2008; Sobacchi et al., 2013; Palagano et al., 2018). The less-frequent “OCL-poor” ARO variant is caused by absence of OCLs due to mutations in *TNFSF11*, which encodes RANKL, or in *TNFRSF11A*, which encodes RANK, the RANKL receptor, that are essential for OCL formation (Sobacchi et al., 2013; Palagano et al., 2018). HSCT from healthy donors can induce the formation of functional OCLs in the patients and significantly improve their clinical presentation (Hashemi Taheri et al., 2015; Orchard et al., 2015; Stepensky et al., 2019; Shapiro et al., 2020). However, because of the genetic heterogeneity of ARO, this treatment is often limited to cases where the underlying genetic mutation does not induce severe symptoms in other tissues, which cannot be corrected by HSCT.

## The R51Q Mutation in Sorting Nexin 10 – a Cause of ARO in Palestinian Communities and Elsewhere

### ARO in Palestine

**FIGURE 2:**
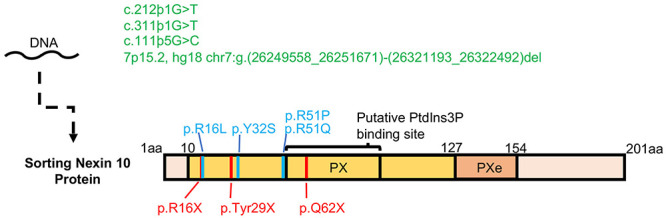
Osteopetrotic mutations in SNX10. Three mutations that affect exon splicing and a large scale deletion in the *SNX10* locus are shown in green. Non-sense mutations leading to an early stop codon, which may produce a highly truncated and potentially unstable protein product are shown in red. Known missense mutations, which may affect the stability of the SNX10 protein or its ability to bind lipids and proteins, are shown in blue. A compound heterozygous nonsense mutation of p.Q62X with p.R16L has also been described. The PX and Pxe (extended PX domain, Xu et al., 2014 Proteins 82, 3483-3489, 2014) are marked in yellow and orange, respectively.

A major source of discovery of genes for AR diseases are consanguineous (inbred) populations (Antonarakis, 2019; Bamshad et al., 2019). Worldwide, ∼10% of marriages are estimated to be consanguineous (Bittles and Black, 2010), but this figure varies considerably between geographical regions. In a manner directly related to the topic discussed here, consanguinity rates are high among Arabs in the Middle East, in particular in Palestine, where 40–50% of marriages are consanguineous. Currently, 18–20% of all Palestinian marriages are between first cousins, a decrease from ∼25% in previous generations (Sharkia et al., 2008, 2016; Zlotogora and Shalev, 2014). Aker et al. (2012) described for the first time a link between ARO and a homozygous missense mutation in *SNX10* in three consanguineous families from the Palestinian village of Karma that is located near the city of Hebron (OMIM 614780). SNX10 belongs to the large family of sorting nexin proteins that, in eukaryotes, numbers over 30 structurally diverse members. All SNX proteins contain a phox-homology (PX) domain through which they bind phospholipids, mainly phosphatidylinositol-3-phosphate (PI3P) that is present in early endosomes. Indeed, a number of individual SNX proteins participate in the sorting of endocytosed proteins to degradation or to recycling, but many of the molecular details and cellular implications of these activities, including in OCLs, remain unknown (Worby and Dixon, 2002; Cullen, 2008; Teasdale and Collins, 2012; Gallon and Cullen, 2015). At 201 amino acids SNX10 is among the smaller SNX family members and, apart from an N-terminal extended PX domain, it lacks any known structural motifs (Cullen, 2008; Xu et al., 2014) (Figure 2).

The initial mutation described by Aker et al. (2012) was a missense mutation in exon 3 (c.152 G/A), leading to the replacement of a highly conserved arginine residue with a glutamine at position 51, which is located within the PX domain. OCLs from patients were fewer in number and smaller than OCLs from healthy controls, and their bone-resorbing activity was significantly inhibited (Aker et al., 2012). Following that study, eight additional families with multiple affected individuals suspected of suffering from ARO were recruited from the village of Karma and are presented here. All these families belonged to the same clan and bore the same family name. Upon interviewing, these families recognized that they most likely shared a common ancestor, but could not trace their relatedness despite frequent consanguineous marriages within each family. Following diagnosis, which was based mainly on clinical examination and on panoramic X-rays, similar findings were observed in affected individuals from all eight families, suggesting a common etiology. The most prominent features observed included early manifestation of disease, dysmorphic features, increased bone density, multiple fractures of legs and ribs, vision problems with either unilateral or bilateral vision loss, enlarged spleen and liver, pancytopenia and recurrent acute infections. The systemic findings suggested that these individuals suffer from the most severe manifestation of osteopetrosis, which is ARO.

**FIGURE 3:**
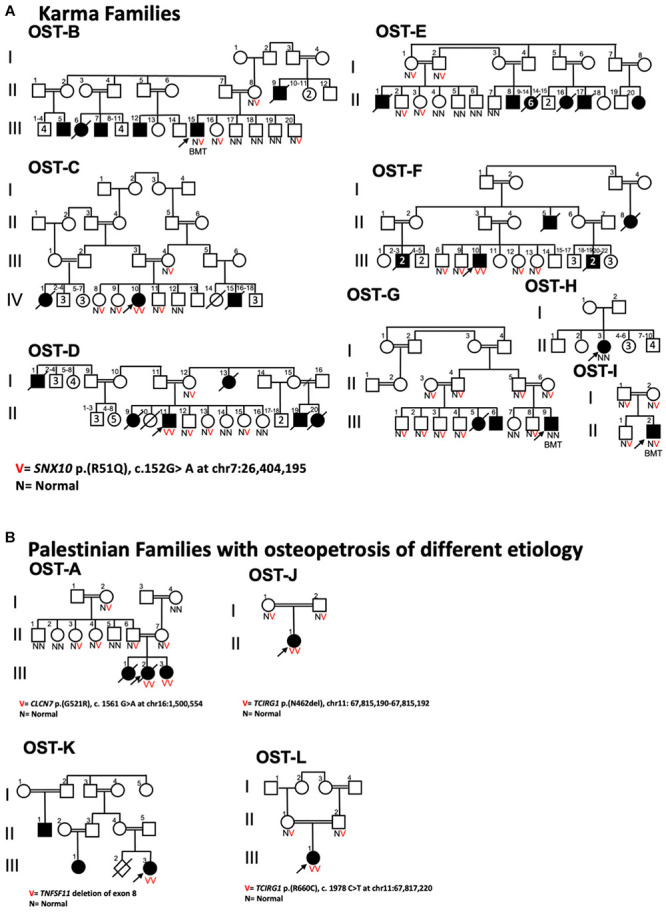
Pedigrees of Palestinian families displaying ARO. **(A)** Karma village families displaying *SNX10* p.R51Q genotype of sampled individuals; arrows indicate probands. Highly consanguineous marital relationships produce offspring with two copies of the mutant *SNX10* allele that segregate with ARO phenotype. B/III-15, G/III-9, and I/II-2 ARO patients underwent hematopoietic stem cell transplantation, and thus their allele compositions reflect those of their unaffected donors. The only exception is the affected individual H/II-3 in OST-H Family, who was wildtype for SNX10_p.R51Q variant as discussed in the text. **(B)** Pedigrees of four Palestinian families from other regions of the West Bank with affected individuals diagnosed with osteopetrosis displaying different genetic mutations. The affected individual of a family from Ramallah (OST-J family) was homozygous for a frameshift mutation (p.N462del) in the *TCIRG1* gene, and the affected individual from the Bethlehem family (OST-L family) was homozygous for a non-synonymous mutation (p.R660C) in the same gene. The affected individual from the family from Yatta (OST-A family) was homozygous for a missense mutation in *ClCN7* gene: *CLCN7* p.G521R. A second family from Ramallah (OST-K family) carries a deletion mutation of exon 8 in *TNFSF11* gene.

Sequencing and segregation analyses confirmed that the *SNX10* p.R51Q variant was present in almost all affected individuals (B/III-15, C/IV-10, D/II-11, E/II-1, F/III-10, G/III-9, and I/II-2) (Figure 3) who were diagnosed with osteopetrosis, and their unaffected parents were heterozygous carriers for this mutation. Following whole exome sequencing, individual H/II-3 in family OST-H, who was wildtype for the *SNX10* p.R51Q variant, was found to be heterozygous for a frameshift mutation (p.C496fs^∗^0, c.1485delC at Chr20:10,629,281) in the *JAG1* gene, which encodes the JAGGED 1 protein. Heterozygous mutations in this gene are known to cause Alagille syndrome (OMIM #118450), whose associated skeletal anomalies may be confused with osteopetrosis.

**FIGURE 4:**
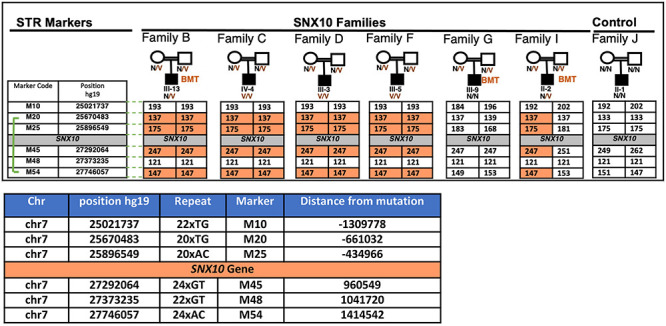
Haplotype analysis of the *SNX10* locus in affected Palestinian families from the village of Karma. Six microsatellite repeat markers that flank the *SNX10* gene, three on either side, were selected and amplified by PCR using fluorescent labeled primers and examined by capillary electrophoresis (CE). Haplotype analyses were analyzed using GeneScan. Selected markers were tested on SNX10 positive families on the affected individual and the parents, one heterozygous sibling (not shown here). Haplotype analysis revealed that all Karma patients who are homozygous for p.R51Q mutation were also homozygous for all studied markers. BMT denotes patients who had undergone hematopoietic stem cell transplantation prior to analysis. Different haplotypes were found in unrelated OST-J Family (Figure 3), as expected. The minimum shared haplotype block (orange) linked to the mutation was found in all the affected *SNX10* patients. For each repeat the following parameters are provided: its position [according to the Human Genome Assembly GRCh37 (hg19), https://www.ncbi.nlm.nih.gov/assembly/GCF_000001405.13/], the length and composition of the nucleotide repeat, and its distance in bps from the R51Q mutation in the SNX10 gene.

Four families from areas of the West Bank outside the village of Karma with affected individuals diagnosed with osteopetrosis were also enrolled in this study (Figure 3). Sanger sequencing of these affected individuals ruled out the *SNX10* p.R51Q mutation as cause for their disease, while whole exome sequencing revealed a different genetic basis for the disease in each case. These mutations all occur in genes that have been linked to ARO (Sobacchi et al., 2013; Palagano et al., 2018), and are described in Figure 3. The results obtained with these four families suggested that the *SNX10* p.R51Q mutation is localized to the village of Karma and could have appeared as a result of founder effect. Haplotype analyses, utilizing six short tandem repeat (STR) markers around the *SNX10* p.R51Q locus, were therefore conducted in order to trace the relatedness among the Karma families. From each family, one affected patient homozygous for the *SNX10* p.(R51Q) mutation, one heterozygous sibling, and both parents were examined. The OST-J family, which hails from a different geographical location and whose ARO is caused by a distinct genetic event (Figure 3), was also screened for the same microsatellite markers as a control. Haplotype analysis revealed that all Karma patients who were homozygous for p.R51Q mutation and who had not undergone HSCT were also homozygous for all the chosen markers, as indicated in Figure 4. The minimum shared haplotype block linked to the mutation was found in all the affected *SNX10* patients, while a different haplotype block was found in the OST-J family. This data provides additional evidence that the shared haplotype block is linked to p.R51Q mutation, and excludes the possibility of sharing it by pure chance in this endogamous population. The shared haplotype block also suggests the p.R51Q mutation in the Karma patients originates in a common founder and was spread in this community by common ancestry. Further studies, in which whole exome sequencing data of 1352 Palestinian individuals from the West Bank and Gaza was examined, revealed only two individuals as carriers of the p.R51Q mutation, corresponding to an estimated allele frequency of 0.00074. In contrast, examination of 3700 individuals from Karma and the surrounding villages identified 89 individuals, belonging to 35 extended families, as heterozygous carriers of this mutation, indicating an allele frequency of 0.024.

### SNX10-Based ARO in Other Geographical Locations

Following the original discovery of the R51Q mutation in SNX10, other mutations in *SNX10*, often in exons 3 and 4, have been linked to this disease in other geographical regions (Mégarbané et al., 2013; Pangrazio et al., 2013; Simanovsky et al., 2016; Stattin et al., 2017; Baer et al., 2019; Kocak et al., 2019; Stepensky et al., 2019) (see Table 1 and Figure 2). Overall, it is estimated that 4.5% of the cases of ARO worldwide are caused by mutations in this gene (Sobacchi et al., 2013; Palagano et al., 2018). Patients with different *SNX10* mutations show symptoms of sclerosis that often also include cranial malformation, secondary neurological defects, vision impairment, and anemia, but usually lack overt immunological symptoms (Pangrazio et al., 2013; Simanovsky et al., 2016; Kocak et al., 2019). Recent evidence also suggests that synonymous mutations, which do not lead to an exchange of an amino acid or truncated protein and previously considered silent, can still cause symptoms of osteopetrosis (Palagano et al., 2017). Mutations leading to an intermediate osteopetrosis form, not as severe as ARO nor as benign as the dominant autosomal form of osteopetrosis, have also been detected in *SNX10* (Stattin et al., 2017; Baer et al., 2019). Although the symptoms of this intermediate disease present during infancy, it does not require urgent intervention. Patients suffering from this variant of *SNX10*-based osteopetrosis, as well as from other forms of intermediate disease, nonetheless accumulate debilitating skeletal complications as they age, and may eventually require blood transfusions and curative treatment (Stepensky et al., 2019). A group of nine *SNX10* intermediate osteopetrosis patients was described in the Swedish county of Västerbotten, which were caused by a splice site mutation in the *SNX10* gene that led to a frameshift and premature termination of translation of the protein product (Stattin et al., 2017). Individuals with this variant of osteopetrosis have survived without HSCT up to the age of 47 years (Stattin et al., 2017).

**TABLE 1:** Osteopetrotic SNX10 mutations.

**Genotype**	**Mutation**	**Effect on SNX10 protein**	**Source**
p.Arg51Gln	Missense	Reduces protein stability/binding	Aker et al. (2012)
p.Arg16Leu	Missense	Disrupts interactions with other proteins	Pangrazio et al. (2013)
p.Tyr32Ser	Missense	Destabilizes internal packing	Pangrazio et al. (2013)
p.Arg51Pro	Missense	Alters the main chain conformation or affects binding to other proteins	Pangrazio et al. (2013)
p.Arg16X	Non-sense	Truncated form of SNX10	Pangrazio et al. (2013)
p.Tyr29X	Non-sense	Truncated form of SNX10	Pangrazio et al. (2013)
p.Gln62X	Non-sense	Truncated form of SNX10	Pangrazio et al. (2013)
p.Arg16Leu and p.Gln62X	Non-sense (compound heterozygous)	Truncated form of SNX10	Pangrazio et al. (2013)
c.212 þ1G>T	Splice site nucleotide change	Impairs the splicing between donor splice sites of exons 4 and 5	Pangrazio et al. (2013)
c.311 þ1G>T	Splice site nucleotide change	Impairs the splicing between donor splice sites of exons 4 and 5	Pangrazio et al. (2013)
c.111 þ5G>C	Splice site nucleotide change	Reduces strength of the donor splice site of exon 3	Pangrazio et al. (2013)
c.43delG	Non-sense	Truncated form of SNX10	Amirfiroozy et al. (2017)
c.284 G>A	Missense?		Shamriz et al. (2017)
7p15.2, hg18 chr7:g.(26249558 _26251671)-(26321193_26322492)del	Deletion	Del including a large region upstream of SNX10 and a partial non-coding region of SNX10	Baer et al. (2019)

Studies conducted during the last decade have taught us that *SNX10*-based osteopetrosis is heterogeneous in nature. Rare *de novo* mutations as well as related patient clusters sharing the same mutation were found, with the severity of the resulting disease linked to the underlying mutation. It is interesting to note that most missense mutations in SNX10 that led to ARO were localized at the beginning of the protein (Table 1); missense mutations that affect more downstream residues remain to be discovered. Absence of such mutations in the population could mean that such mutations do not cause disease or, conversely, that the severity of the disease precludes survival.

## Insights From Model Systems Into the Molecular Role of SNX10 in Ocls

The known functions of SNX proteins suggest that SNX10 regulates osteoclastogenesis by controlling cellular membrane trafficking, and that the underlying mechanism can be characterized through cellular and molecular studies of SNX10-based ARO. While some of these studies can be performed in OCLs grown from human monocytes, patients of ARO caused by mutations in *SNX10* are rare, and most are treated with HSCT. A significant part of our knowledge was therefore obtained from model systems, such as genetically manipulated cells or mice. Notably, studies of this nature have been most revealing, yet interpretation of data obtained from these model systems should be done with obvious care.

### Studies of Cells From Human Patients

In the original study that linked SNX10 to ARO, Aker et al. (2012) showed that OCLs prepared *in vitro* from mononuclear leukocytes of ARO patients who were homozygous for R51Q SNX10 were inactive in *in vitro* assays. The cells also contained large vacuoles (Aker et al., 2012), which may be caused by defects in the sorting and fusion of cellular vesicles. Subsequent studies of the intermediate ARO cluster of cases from Västerbotten revealed that OCLs prepared from peripheral blood of patients contained defective RBs, and the cells did not resorb bone in *in vitro* assays. The cells were also three- to four-fold larger than OCLs from healthy individuals, a finding attributed to increased spreading (Stattin et al., 2017). Induced pluripotent stem cells were more recently produced from a patient carrying the Västerbotten mutation (Xu et al., 2017). This approach may help bypass the difficulties in obtaining hematopoietic cells from patients and will likely further advance our understanding of how this mutation affects SNX10 and its function in OCLs.

### Mouse Models of SNX10 Mutations in ARO

Several mouse models in which the *Snx10* gene has been targeted globally or specifically in OCLs have been described (Table 2). Ye et al. (2015) presented global knockdown (KD) mice, in which a selection cassette was inserted into intron 3 of *Snx10* and inhibited its expression, resulting in a drop of 86% in *Snx10* mRNA levels in bone samples. The phenotype of these mice was severe and combined osteopetrosis and rickets. Homozygous mice exhibited massive increases in bone mass, absent teeth and severely retarded growth, and survived up to the age of 3–4 weeks (Ye et al., 2015). SNX10 KD OCLs were nearly inactive *in vitro* due to presence of rudimentary RBs and an inability to acidify the extracellular space, processes that rely on proper vesicular trafficking and membranal function. Paradoxically, the serum levels of collagen I C-telopeptides (CTX), which are interpreted as a clinical indicator of OCL mediated bone resorption *in vivo*, were significantly increased in these mice (Ye et al., 2015). Similar observations were made in other models of osteopetrosis despite demonstrably low OCL activity (e.g., Neutzsky-Wulff et al., 2008; Stein et al., 2020), indicating that circulating CTX levels might not accurately reflect *in vivo* OCL activity in these cases. Bone formation, as assessed by the clinical marker P1NP, was unaltered.

**TABLE 2:** Key cellular phenotypes in OCLs from “OCL-rich” ARO mouse models.

**Gene**	***Snx10***	***Clcn7***	***Ostm1***	***Tcirg1***	***Plekhm1***
Protein	SNX10 Sorting nexin 10	CLCN7 Chloride voltage-gated channel 7 (chloride/proton antiporter)	OSTM1 Osteoclastogenesis associated transmembrane protein 1 (beta subunit of CLCN7)	ATP6v0a3, Atp6i T cell immune regulator 1, ATPase H + transporting V0 subunit a3 (a3 subunit of the H^+^-ATPase)	PLEKHM1 Plekstrin homology and RUN domain containing M1
Mouse models	R51Q SNX10 knock-in, whole body (Stein et al., 2020; Barnea-Zohar et al., 2021). Conditional deletion of exons 4, 5 (Ye et al., 2015).	Whole-body knockout (Kornak et al., 2001; Kasper et al., 2005; Neutzsky-Wulff et al., 2008). Conditional deletion of exons 12, 13 (Wartosch et al., 2009)^2^.	Gray lethal (gl) mice (a naturally occurring mutant of *Ostm1*) (Rajapurohitam et al., 2001; Chalhoub et al., 2003; Lange et al., 2006; Héraud et al., 2014). Conditional in-frame deletion of exon 5 in *Ostm1* (Pata and Vacher, 2018).	Osteosclerotic (Oc) mice (a naturally occurring mutant of *Tcirg1*) (Ito et al., 2001; Schinke et al., 2009). Whole-body knockout of exons 2–5 (Li et al., 1999), conditional deletion of exons 14–20 (Sun-Wada et al., 2009).	Conditional deletion of exon 3 (Fujiwara et al., 2016). Gene-trap disruption of intron 1 (Brommage et al., 2014).
Bone phenotype	Osteopetrosis, growth retardation, no tooth eruption, death at 6–7 weeks (Ye et al., 2015; Stein et al., 2020). Rickets (whole-body Snx10 KD) (Ye et al., 2015).	Osteopetrosis, no rickets, growth retardation, no tooth eruption, death at 6–7 weeks (Kornak et al., 2001; Kasper et al., 2005; Neutzsky-Wulff et al., 2008).	Osteopetrosis, growth retardation, no tooth eruption, death at 3–4 weeks (Rajapurohitam et al., 2001; Chalhoub et al., 2003; Lange et al., 2006; Pata and Vacher, 2018).	Osteopetrosis, osteopetrorickets, growth retardation, no tooth eruption, death at 4–5 weeks (Li et al., 1999; Ito et al., 2001; Schinke et al., 2009; Sun-Wada et al., 2009).	Normal development, teeth present, increased trabecular bone mass (Fujiwara et al., 2016). No bone phenotype (Brommage et al., 2014).
TRAP-positive OCLs present	Yes (*in vivo*/*in vitro*)^1^ (Ye et al., 2015; Stein et al., 2020; Barnea-Zohar et al., 2021).	Yes (*in vivo*/*in vitro*) (Kornak et al., 2001; Neutzsky-Wulff et al., 2008).	Yes. Increased OCL numbers (*in vivo*), TRAP staining *in vivo*/*in vitro* (Rajapurohitam et al., 2001; Pata and Vacher, 2018).	Yes. Increased OCL numbers (*in vivo*) (Li et al., 1999; Ito et al., 2001).	Yes (*in vitro*/*in vivo*). TRAP staining: *in vitro*, perinuclear accumulation (Fujiwara et al., 2016).
OCL activity	Inactive (*in vitro*) (Ye et al., 2015; Stein et al., 2020).	Inactive (*in vivo*/*in vitro*) (Kornak et al., 2001; Neutzsky-Wulff et al., 2008).	Virtually inactive (*in vitro*) (Rajapurohitam et al., 2001; Pata and Vacher, 2018).	Inactive (*in vivo*/*in vitro*) (Li et al., 1999).	Nearly absent (*in vitro*) (Fujiwara et al., 2016).
Lysosomes		Present, normal pH (Kornak et al., 2001; Kasper et al., 2005).	Present, normal pH, dispersed organization (Pata and Vacher, 2018).	Present (Li et al., 1999).	Present, abnormal perinuclear positioning (Fujiwara et al., 2016).
Ruffled border	Absent (Stein et al., 2020) Rudimentary (Ye et al., 2015).	Nearly absent (Kornak et al., 2001).	Underdeveloped (Rajapurohitam et al., 2001).	Absent (Ito et al., 2001).	Absent (Fujiwara et al., 2016).
Extracellular acidification	Absent (Ye et al., 2015; Stein et al., 2020).	Absent (*in vitro*) (Kornak et al., 2001).	Absent (*in vitro*) (Pata and Vacher, 2018).	Absent (*in vitro*) (Li et al., 1999).	
Endocytosis	Impaired (Ye et al., 2015; Barnea-Zohar et al., 2021).				Normal
OCL shape/size	*In vitro*: Gigantic OCLs, due to continuous cell-cell fusion. (Barnea-Zohar et al., 2021).	*In vivo*: abnormally elongated (Kornak et al., 2001), large OCLs (Neutzsky-Wulff et al., 2008).	*In vitro*: elongated. (Rajapurohitam et al., 2001), larger than WT, due to increased NFATc1 activity-? (Pata and Vacher, 2018).		Abnormal, less round OCL shape, normal size, *in vitro* (Fujiwara et al., 2016).
Comments		Accumulation of lysosomal storage materials in forebrain neurons and proximal tubule cells (Kasper et al., 2005; Wartosch et al., 2009).	Defective exocytosis of TRAP and Cathepsin K in OCLs (Pata and Vacher, 2018). Normal lysosomes, abnormal autophagosomes in neurons (Héraud et al., 2014).		Inactive exocytosis (Fujiwara et al., 2016). Incisor absent (ia) rats, in which *Plekhm1* is disrupted, exhibit age-dependent osteopetrosis and lack teeth. Their OCLs are nearly inactive and lack ruffled borders (Reinholt et al., 1999; McDonald et al., 2011).

*^1^*In vitro*: Determined in OCLs cultured on glass/plastic/bone/dentine from mice. *in vivo*: As observed in animals or directly in tissue samples collected from them.*

*^2^Whole-body E245A CLCN7 mice, in which CLCN7 was modified from a Cl^–^/H^+^ exchanger into an uncoupled Cl- conductor, display severe growth retardation and osteopetrosis and their OCLs display underdeveloped RBs, but these phenotypes are less severe than in whole body KO of CLCN7 (Weinert et al., 2010). Whole-body E312A CLCN7 mice, in which CLCN7 is transport-deficient, display phenotypes similar to whole-body CLCN7 knockouts (Weinert et al., 2014).*

Clear indications of rickets were detected in homozygous SNX10 KD mice, in the form decreased bone mineral density and bone mineral content values, presence of non-mineralized osteoid on trabecular surfaces, and metaphyseal fraying and cupping in femurs and tibias (Ye et al., 2015). Indeed, the mice were hypocalcemic and exhibited increased serum levels of PTH and reduced levels of 25-hydroxy-vitamin D3. Further studies suggested that this is caused by a severe reduction in the expression of SNX10 in gastric epithelial cells, which resulted in elevated gastric pH and stomach necrosis that led to reduced uptake of dietary calcium. Dietary supplementation with calcium gluconate resolved the rachitic phenotypes of these mice.

The same study also described mice in which SNX10 was targeted specifically in OCLs (SNX10 OCL mice), by crossing mice carrying a floxed allele of *Snx*10 that deleted exons 4 and 5 with mice expressing Cathepsin-K-Cre (Ye et al., 2015). While SNX10 OCL mice were osteopetrotic and lacked teeth, their overall phenotype was less severe than SNX10 KD mice: the mice exhibited mild growth retardation by 3 weeks of age, but were reported to survive and did not display rachitic or gastric abnormalities (Ye et al., 2015). An additional strain of mice in which the *Snx10* gene was targeted and which are also osteopetrotic has been described (Zhou et al., 2016). OCLs from these mice appear to resorb bone less well than WT OCLs and to express reduced amounts of beta 3 integrin, TRAP, Cathepsin K, MMP9, and possibly NFATc1. Unfortunately, the genetic manipulation that gave rise to these mice has not been described, preventing a more precise understanding of this model.

Stein and colleagues Stein et al. (2020) created a whole-body knock-in R51Q SNX10 mouse, modeling the first mutation in this protein that was linked to ARO (Aker et al., 2012). Homozygous R51Q SNX10 mice were massively osteopetrotic, lacked teeth, exhibited significant developmental delay at the age of 4 weeks, and generally did not survive past the age of 6–8 weeks (Stein et al., 2020). OCLs from these mice lacked RBs and could not acidify the cell-bone interface, in agreement with their complete lack of activity in *in vitro* pit resorption assays. R51Q SNX10 OCLs also stained poorly for TRAP, appeared to be less adherent, and were noticeably more fragile and shorter-lived than their wild-type or heterozygous counterparts (Barnea-Zohar et al., 2021). Levels of CTX in circulation were elevated in these mice despite clear OCL inactivity (Stein et al., 2020), similar to the SNX10 KD mice (Ye et al., 2015). Osteoblast numbers were increased in R51Q SNX10 mice, but their bone formation rates, serum levels of P1NP, and *in vitro* differentiation of OBs were normal, indicating that their osteopetrotic phenotype was due to OCL dysfunction (Stein et al., 2020). This result is consistent with the very low expression of SNX10 in OBs relative to OCLs (Stein et al., 2020).

The bone-related phenotypes of the R51Q SNX10 knock-in mice and the SNX10 KD mice are overall similar and confirm a critical role for this protein in OCLs and in bone degradation. Nonetheless, R51Q SNX10 mice are not rachitic, an observation that agrees with the symptoms of human patients of SNX10-based ARO (Palagano et al., 2018). The rickets phenotype of SNX10-KD mice has been linked to dysfunction of gastric epithelial cells (Ye et al., 2015), which apparently does not occur in the R51Q SNX10 mice. This distinction may be caused by the differences between both model systems: the R51Q SNX10 mutation results in production of a mutant protein whose expression levels and functions in gastric epithelial cell are unknown, while the SNX10 KD model decreases expression of the WT protein to low levels (Ye et al., 2015; Stein et al., 2020).

### Cell-Based Studies

Studies of SNX proteins in cultured cell lines and in primary cells suggest that SNX10 functions in regulating vesicular trafficking in cells. In particular, the PX domain of SNX10, like those of most other SNX proteins (Teasdale and Collins, 2012), predominantly binds PI3P, which is found in early endosomes; SNX10 also binds phosphatidylinositol-3,5-diphosphate (PI(3,5)P_2_) that is prominent in late endosomes (Haucke, 2005; Wallroth and Haucke, 2018; Barnea-Zohar et al., 2021). In agreement with these findings, studies in RAW264.7 cells indicated that SNX10 binds the lipid kinase PIKfyve, which phosphorylates PI3P into PI(3,5)2P, and both PIKfyve and SNX10 co-fractionate in these cells together with EEA1, a marker of early endosomes (Battaglino et al., 2019; Sultana et al., 2020). Qin et al. (2006) showed that SNX10 co-localizes with endosomal markers also in HeLa cells. Expression of exogenous SNX10, but not of several other SNX family members, in these cells led to accumulation of large vacuoles that required presence of both the PX domain of SNX10 and its C terminal sequence (Qin et al., 2006). These findings suggest that SNX10 performs a role in early endosomes, and consequently the effects of its disruption might be mediated by this cellular compartment.

SNX10 has also been shown to affect lysosomes and autophagosomes. SNX10 fulfills an essential role in chaperone-mediated autophagy (CMA), a process in which specific soluble cytosolic proteins are delivered to lysosomes for degradation in an autophagosome-independent manner. In CMA, target proteins associate initially with heat shock protein 70 (Hsc70), and the resulting complex binds LAMP2A at the lysosomal membrane and is translocated into the lysosomal lumen for degradation (Kaushik and Cuervo, 2018). SNX10 destabilizes LAMP2A, most likely by promoting maturation of cathepsin A that plays a major role in LAMP2A degradation (You et al., 2018; Zhang et al., 2018). Loss of SNX10 stabilizes LAMP2A and increases cellular CMA activity, which is associated with increased AMPK signaling in livers and in primary hepatocytes of SNX10 knockout mice (You et al., 2018; Zhang et al., 2018). Studies in the human carcinoma cell line HCT116 indicated that SNX10 interacts through specific residues located in its C-terminal sequence with the lysosomal protein LAMP1 (Zhang et al., 2018, 2020); this interaction was reported to be essential for inducing degradation of LAMP2A by a yet-unknown mechanism (Zhang et al., 2018).

In additional studies, expression of SNX10 was upregulated during autophagy in HCT116 and in various other cell lines (Zhang et al., 2020). SNX10 binds, through its C-terminus, the ATG5 protein in these cells, thereby associating also with the ATG12-ATG5 conjugate that is located on the surface of autophagosomes (Zhang et al., 2020) and which plays important roles in the formation and maturation of autophagosomes and in their fusion with lysosomes (Chen et al., 2012). Further studies indicated that loss of SNX10 or mutation of specific C-terminal residues through which it binds ATG5 or LAMP1 inhibited autophagosome-lysosome fusion in HCT116 cells. According to the model that emerges from these findings, a population of SNX10 molecules associates with LAMP1 and with lysosomes, while other SNX10 molecules associate with the ATG12-ATG5 conjugate and with autophagosomes. Interactions between SNX10 molecules may bring these two organelles in close proximity and may promote their fusion (Zhang et al., 2020). Finally, SNX10 also enhances the bactericidal activity of macrophages by promoting acidification of their phagosomes, which occurs via phagosome maturation and fusion with lysosomes (Lou et al., 2017). Maturation of phagosomes and endosomes occurs by similar mechanisms, hence these studies further strengthen the conceptual link between SNX10 and early endosomes and the effect that this may have on lysosomes.

**FIGURE 5:**
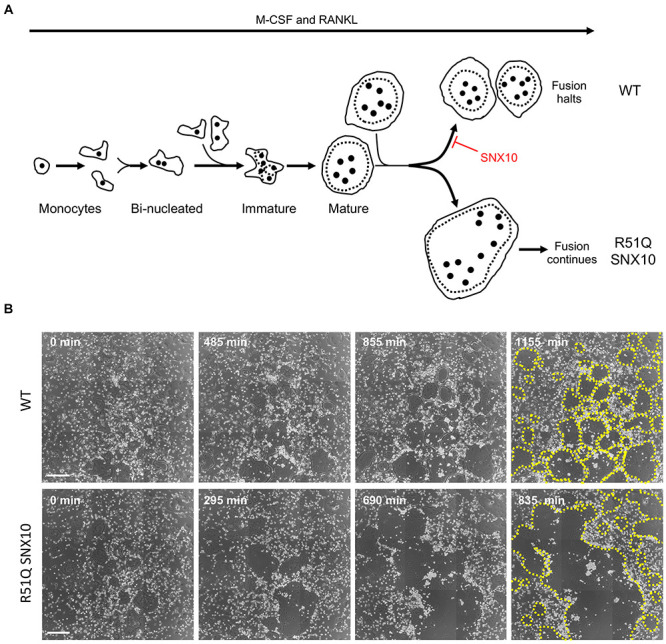
Uncontrolled fusion of mature R51Q SNX10 OCLs. **(A)** Outline of osteoclastogenesis in culture, similar to Figure 1A. Top: WT mature OCLs do not fuse with each other. Bottom: Mature R51Q SNX10 OCLs readily fuse with each other and continue to fuse with other mature OCLs. **(B)** Phase light microscopy images of cultures of WT (top) and R51Q SNX10 (bottom) spleen-derived monocytes undergoing osteoclastic differentiation on glass coverslips in the presence of M-CSF and RANKL. Note development of mature, round WT OCLs that remain juxtaposed with each other, vs. continuous fusion that generates giant R51Q SNX10 OCLs (Barnea-Zohar et al., 2021). Times are noted from start of imaging; OCL boundaries are indicated by dashed yellow lines in the final image of each series. Each image is a composite of nine smaller images. Scale bars: 250 μm.

However, the effects of SNX10 are evident also in other cellular compartments, in which SNX10 is not known to function. One prominent example concerns OCL fusion, a process that initiates at the plasma membrane. When grown *in vitro* on mineralized or non-mineralized surfaces, R51Q SNX10 OCLs fuse repeatedly and continuously to form gigantic OCLs that can become 10- to 100-fold larger than controls (Barnea-Zohar et al., 2021). Examination of the fusion of mutant OCLs in culture by live-cell imaging revealed widespread fusion between mature OCLs, a fusion modality that was rarely observed among WT OCLs (Figure 5). In particular, mature juxtaposed R51Q SNX10 OCLs readily fused with each other and continued to fuse with additional mature OCLs, stopping only when no more fusion partners were available or when the cells died. In contrast, mature juxtaposed WT OCLs did not fuse with each other, and fusion ceased when such cells became surrounded by other similarly mature OCLs (Figure 5) (Barnea-Zohar et al., 2021). Molecularly, the R51Q mutation, which is located within the lipid-biding PX domain of SNX10, leads to the loss of PI3P binding and destabilizes the protein, severely reducing its levels in the cells (Barnea-Zohar et al., 2021). In agreement with this notion, expression of WT SNX10 rescued the deregulated fusion phenotype of primary R51Q SNX10 OCLs, while CRISPR-mediated knockout of the *Snx10* gene induced deregulated fusion in RAW264.7 cells (Barnea-Zohar et al., 2021). Taken together, these findings indicate that the hyper-fusion phenotype of R51Q SNX10 OCLs is induced by loss of function of SNX10. From a broader perspective, these findings indicate that fusion between mature OCLs is actively down-regulated by a cell-autonomous, genetically regulated mechanism, which requires SNX10 and is disrupted by the R51Q mutation. We note that in another study, knockdown of SNX10 by lentiviral-delivered shRNA prevented differentiation of RAW264.7 cells into mature OCLs (Zhu et al., 2012). The different results of this study vs. the CRISPR-mediated knockout of *Snx10* in RAW264.7 cells (Barnea-Zohar et al., 2021) may arise from distinct effects of acute (shRNA) vs. long-term, constitutive (CRISPR) elimination of *Snx10*, or from incomplete down-regulation of *Snx10* by shRNA.

How might R51Q SNX10 promote fusion between mature OCLs? Cell-cell fusion initiates when fusogens, specialized fusion-promoting molecules that are present at the cell surface, induce fusion of juxtaposed cells (Oren-Suissa and Podbilewicz, 2007; Chernomordik and Kozlov, 2008; Martens and McMahon, 2008; Helming and Gordon, 2009; Willkomm and Bloch, 2015; Brukman et al., 2019). Studies of OCLs from R51Q SNX10 and from SNX10KD mice revealed that internalization of dextran, which occurs by both fluid-phase and receptor-mediated endocytosis (Pustylnikov et al., 2014), is significantly reduced in these cells (Ye et al., 2015; Barnea-Zohar et al., 2021). Inhibition of endocytosis could result in aberrant retention of fusogens or of other fusion-promoting molecules at the cell surface, thereby enabling continuous fusion. In support of this proposal we note that endocytosis was shown to inhibit fusion of epidermal cells in *C. elegans* by selectively removing the fusogen EFF-1 from their surface (Smurova and Podbilewicz, 2016). However, endocytosis was also shown to promote fusion of monocytes in the early stages of osteoclastogenesis (Shin et al., 2014), suggesting that its role in cell-cell fusion might depend on the type and on the developmental stage of the fusing cells. This proposal can be tested directly by deep differential analysis of the plasma membrane proteome of R51Q SNX10 OCLs. Such analyses would help to identify putative fusion-promoting proteins that are downregulated in WT OCLs but are retained in the mutant cells.

A second example of a defect in R51Q SNX10 OCLs in a cellular compartment in which SNX10 does not function directly is the absence of the RB in mutant OCLs. The presence and function of SNX10 in autophagosomes, which fuse with lysosomes, and in lysosomes themselves suggest that loss of SNX10 or its R51Q mutant may adversely affect these compartments, thereby preventing subsequent fusion of lysosomes with the ventral cell membrane. Such an event would block formation of the RB and lead to OCL inactivity, as was indeed observed in R51Q SNX10 and in SNX10-KD mice (Ye et al., 2015; Stein et al., 2020; Barnea-Zohar et al., 2021). Collectively, the above results lead us to propose that the R51Q mutation in SNX10 leads to a loss-of-function of this protein that disrupts the formation or function of endosomes and lysosomes. The central role of these cellular compartments in vesicular trafficking results in disruption of events along the plasma membrane-to-RB axis, including some in which SNX10 is not known to play a direct role.

## Discussion

**FIGURE 6:**
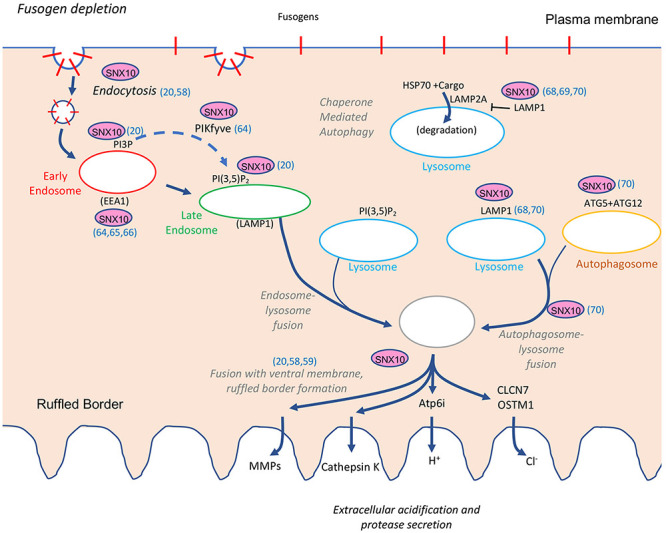
Overview of the known functions of SNX10 along the plasma membrane-to-ruffled border axis. Functions or abilities indicated include support of endocytosis (references Ye et al., 2015 and Barnea-Zohar et al., 2021 noted in the figure), binding to PI3P and PI(3,5)P_2_ (Barnea-Zohar et al., 2021), association with PIKfyve, EEA1, and early endosomal markers (Qin et al., 2006; Battaglino et al., 2019; Sultana et al., 2020), inhibition of chaperone mediated autophagy (You et al., 2018; Zhang et al., 2018, 2020), association with LAMP1 and ATG5 and promotion of lysosome-autophagosome fusion (Zhang et al., 2018, 2020), and ruffled border formation (Ye et al., 2015; Stein et al., 2020; Barnea-Zohar et al., 2021). See the section “Cell-Based Studies,” for additional details. PI3P, phosphatidylinositol-3-phosphate; PI(3,5)P_2_, phosphatidylinositol-3,5-diphosphate; Atp6i, the a3 subunit of the V0 domain of the OCL ATP-dependent proton pump; CLCN7, voltage-gated chloride/proton antiporter; OSTM1, beta subunit of the voltage-gated chloride/proton antiporter; CathK, cathepsin K; MMPs, matrix metalloproteases.

In this article we have explored and discussed possible mechanisms whereby SNX10 might act as a key regulator of bone homeostasis via its involvement in OCL development and function. Starting with a specific monogenic disease (ARO in the Palestinian community) and an associated mutation (R51Q in SNX10) we discussed here both the population genetics background and the relevant mechanistic insights that may explain why individuals who are homozygous for this mutation develop osteopetrosis. While the direct role of SNX10 in OCL function is still unknown, nearly all the manifestations of the R51Q mutation are related to aberrant membrane properties, including lipid interaction specificity, cell fusion, matrix adhesion, endocytosis, RB formation, and acidification of the resorption pit. Several of these phenotypes, e.g., deregulated cell fusion and RB formation, affect processes that SNX10 is not known to regulate directly, and most likely result from the broader disruption to the plasma membrane-RB axis (Figure 6).

The observation that severe membranal trafficking defects, such as those described here, are nonetheless compatible with embryo survival deserves some consideration. The phenotypes of the whole-body SNX10-KD and R51Q SNX10 mice are indeed severe, yet the mice are born and survive for several weeks. A possible explanation for this may be obtained from data from the Human Protein Atlas^1^ that indicate that while SNX10 levels in OCLs are high, its expression in other cell types – and likely the consequences of its mutation – is not ubiquitous. Moreover, possible functional redundancies between SNX10 and other members of the SNX protein family may enable other cells and tissues, in which SNX10 is expressed, to escape the effects of its mutation.

It is noteworthy that most cases of ARO that are not SNX10-related, whose genetic basis is known, are caused by mutations in genes that also perform membrane regulatory functions in OCLs (Sobacchi et al., 2013; Palagano et al., 2018) (Table 2). Among these is the *TCIRG1* gene, which encodes the α3 subunit of the V0 domain of the ATP-dependent proton pump, the V-ATPase, mutations in which cause approximately half of ARO cases globally. Importantly, the lysosomal V-ATPase also participates in vesicle sorting and formation of RBs in addition to its role in acidifying lysosomes and the extracellular space (Coxon and Taylor, 2008; Sobacchi et al., 2013; Palagano et al., 2018). Also included are the *CLCN7* gene, whose product is the voltage-gated chloride/proton antiporter of late endosomes and lysosomes, as well as *OSTM1*, which encodes the beta subunit of this antiporter. Mutations in these two genes account for approximately 17.5% and 5%, respectively, of ARO cases. A small number of ARO cases are also caused by mutations in *PLEKHM1*, whose protein product functions in lysosomal trafficking and autophagosome-lysosome fusion, while 4.5% of ARO cases are linked to mutations in *SNX10*, whose relevance to membranal trafficking in OCLs has been discussed here (Sobacchi et al., 2013; Palagano et al., 2018). The finding that the major sub-types of ARO arise mostly by disruptions in genes that function in membranal trafficking in OCLs emphasizes the critical importance of this process in promoting properly regulated osteoclastogenesis and OCL resorption activity. Moreover, many of the mouse models developed to study mutations in these ARO-associated genes display several common phenotypes that include, in addition to massive osteopetrosis, absent or under-developed RBs, an inability to acidify the OCL-bone interface, reduced or absent bone-resorbing activity, and altered OCL shape or size (e.g., Kornak et al., 2001; Rajapurohitam et al., 2001; Kasper et al., 2005; Henriksen et al., 2006; Lange et al., 2006; Neutzsky-Wulff et al., 2008; Wartosch et al., 2009; Weinert et al., 2010; Héraud et al., 2014; Pata and Vacher, 2018) (Table 2). It is therefore tempting to propose that mutations in ARO-associated genes disrupt specific molecular functions that affect membrane and vesicular trafficking, leading to a wider but similar disruption of the membrane-to-RB axis and to a common set of phenotypes in OCLs. In this context it is of interest to examine OCLs from the various ARO models side by side and compare the precise details and characteristics of their cellular phenotypes. Uncovering these details is critical for obtaining a more complete understanding of OCL biology in particular, and of cellular membrane homeostasis and regulation of membrane trafficking and fusion in general. Furthermore, such information is important for devising novel therapeutic strategies for ARO and other diseases, such as osteoporosis and cancer-related bone loss, in which OCLs are known to play prominent roles. In light of the scarcity of ARO and the resulting small number of cases that can be studied in depth, it is reasonable to assume that studies in cell and animal models will continue to provide important mechanistic insights into these issues.

## Materials and Methods

### Haplotype Analysis Using Microsatellite Markers

Six short tandem repeat (STR) markers around the SNX10_p.R51Q locus (Figure 4) were selected. Primer pairs were designed for amplification of the markers using the Hemi-NeSTR website with default parameters^2^. Each PCR product was mixed with 0.25 μl GeneScan^TM^ 500 ROX^TM^ Dye, and capillary electrophoresis was carried on an Applied Biosystems 3500 Genetic Analyzer (Thermo Fisher Scientific). Gene Mapper software (V5.2) was utilized to call and extract the genotypes from the electropherograms generated by the 3130XL Genetic Analyzer. Haplotypes were constructed manually.

### Whole-Exome Sequencing

Whole exome sequencing was performed at the Hereditary Research Laboratory, Bethlehem University, using Illumina’s NextSeq^TM^ 500 Sequencing System. DNA libraries were prepared using two preparation kits: the Illumina^®^ TruSeq^TM^ DNA Sample Prep Kit, or Nextera^TM^ Flex for Enrichment Prep Kit. Following sequencing, reads were aligned to the reference human genome (hg19) using the Burrows-Wheeler (BWA) aligner. Prior to variant calling by the Genome Analysis Toolkit^3^, mapped reads (BAM format) were re-processed by removing PCR duplicates, realigning around indels, and recalibrating base quality. The final list of variants was annotated by ANNOVAR^4^ (Wang et al., 2010) using several databases of minor allele frequency including gnomAD^5^ and PopFreqMax, as well as variant effect predictors such as SIFT (Sorting Intolerant from Tolerant^6^), PolyPhen-2^7^ and REVEL (Ioannidis et al., 2016). Candidate variants were validated by Sanger sequencing and then tested for co-segregation with the phenotype in additional family members.

### Culture of Primary OCLs

Culture of primary OCLs was performed as described in Barnea-Zohar et al. (2021). In brief, spleens from mice aged 4–8 weeks were dissociated into α-Minimal Eagle’s Medium (α-MEM; Sigma-Aldrich, St. Louis, MO, United States). Following lysis of erythrocytes, 2 × 10^6^ (WT) or 1 × 10^6^ (R51Q SNX10) cells were seeded in 24-well plates. Cells were cultured in complete OCL medium [α-MEM containing 10% fetal calf serum, 2 mM glutamine, 50 units/ml penicillin, 50 μg/ml streptomycin, and M-CSF (20 ng/ml, Peprotech, Rehovot, Israel) for 2 days, after which RANKL (20 ng/ml, R&D Systems, Minneapolis, MN, United States)] was added. Cells were grown at 37°C in 5% CO_2_ for a total of 5–7 days with daily changes of medium. Time-lapse images were acquired with an automated inverted microscope (DeltaVision Elite system IX71 with Resolve3D software modulus; Applied Precision, Inc., GE Healthcare, Issaquah, WA, United States) using a 10×/0.30 air objective (Olympus, Tokyo, Japan). The cells were maintained at 37°C with a 5% CO_2_ humidified atmosphere throughout the imaging process.

### Ethics Board Approval

The human experiments described were approved by the Institutional Review Board of the Istishari Arab Hospital, Ramallah, Palestine. All family members gave their consent to participate in the study. All mouse experiments were approved by the Weizmann Institute IACUC and were conducted in accordance with Israeli law.

## Data Availability Statement

The original contributions presented in the study are included in the article/supplementary material, further inquiries can be directed to the corresponding authors. The data presented in the study are deposited in the ClinVar repository, accession numbers: (1) NC_000018.10:g.60033942_60033993del (TNFRSF11A): SCV001571688/RCV001374875.1, (2) NM_001-199835.1(SNX10):c.152G>A (p.Arg51Gln): SCV001571689/RC-V000033149.3, (3) NM_001287.6(CLCN7):c.1561G>A (p.Gly521Arg): SCV001572330/RCV001375477.1, (4) NM_000-214.3(JAG1):c.1485del (p.Cys496fs): SCV001572331/RCV0013-75478.1, (5) NM_006019.4(TCIRG1):c.1384_1386del (p.Asn462del): SCV001572332/RCV001375479.1, and (6) NM_00-6019.4(TCIRG1):c.1978C>T (p.Arg660Cys): SCV0015723-33/RCV001375480.1

## Ethics Statement

The studies involving human participants were reviewed and approved by Institutional Review Board of the Istishari Arab Hospital, Ramallah, Palestine. Written informed consent to participate in this study was provided by the participants’ legal guardian/next of kin.

## Author Contributions

AE, MK, JT, and BG wrote the manuscript, secured funding, and performed the analysis. MSt and GR wrote the manuscript, collected the data, and performed the analysis. MB-Z, SW-K, NR, MSh, and JS collected the data and performed the analysis. All authors contributed to the article and approved the submitted version.

## Conflict of Interest

The authors declare that the research was conducted in the absence of any commercial or financial relationships that could be construed as a potential conflict of interest.

## Footnotes

1 www.proteinatlas.org

2 http://mpeters.net/primer.html

3 https://gatk.broadinstitute.org

4 http://annovar.openbioinformatics.org/

5 https://gnomad.broadinstitute.org

6 http://sift.bii.a-star.edu.sg

7 http://genetics.bwh.harvard.edu/pph2/

## Abbreviations

ARO: autosomal recessive osteopetrosis
HSCT: hematopoietic stem cell transplantation
M-CSF: macrophage colony-stimulating factor
OB: osteoblast
OCL: osteoclast
RANKL: receptor activator of NFkB ligand
RB: ruffled border
R51Q: arginine-to-glutamine mutation at position 51
SNX10: sorting nexin 10
SZ: sealing zone.
